# Varicella zoster virus-associated non-necrotizing retinitis: case report

**DOI:** 10.1186/s12348-023-00353-2

**Published:** 2023-07-06

**Authors:** Maamouri Rym, Houman Yasmine, Abdelaziz Marwa, Helal Imen, Cheour Monia

**Affiliations:** 1 Department of Ophthalmology, Habib Thameur Hospital, 3, Rue Ali Ben Ayed, 1089 Montfleury, Tunis Tunisia; 2 Department of Pathology, Habib Thameur Hospital, Boulevard du 9 Avril 1938, 1006 Tunis, Tunisia

**Keywords:** Non-necrotizing herpetic retinitis, Varicella zoster virus, Polymerase chain reaction, Case report

## Abstract

**Purpose:**

To describe clinical features and the course of a case of non-necrotizing herpetic retinitis secondary to Varicella zoster virus (VZV).

**Materiel and methods:**

A single case report documented with multimodal imaging.

**Results:**

A 52-year-old female patient with a past medical history of diabetes mellitus who presented with painful red right eye (OD). Ophthalmic examination showed perilimbal conjunctival nodule, granulomatous anterior uveitis, sectoral iris atrophy and increased intraocular pressure. Fundus examination in OD revealed posterior multifocal retinitis. Left eye examination was unremarkable. Polymerase chain reaction (PCR) of aqueous humor sample confirmed the presence of VZV DNA. Systemic antiviral therapy allowed the improvement of intraocular inflammation and disappearance of the retinal non necrotizing retinitis after one year of regular follow-up.

**Conclusion:**

Non-necrotizing retinitis is an underdiagnosed form of VZV ocular infection.

## Introduction

Viral infections due to *Herpesviridae* are common worldwide. Among the spectrum of viral retinitis, acute retinal necrosis (ARN), progressive outer retinal necrosis (PORN), and Cytomegalovirus (CMV) retinitis are the most severe and sight threatening viral infections [1]. Besides ARN, PORN and CMV retinitis; viral retinitis can occur in atypical clinical forms. This uncommon and atypical disease entities can be entitled as non-necrotizing herpetic retinitis (NNHR) which was first reported in 2003 by Bodaghi and colleagues [2]. The purpose of this article is to describe the clinical characteristics of an atypical form of varicella zoster virus (VZV)-associated non-necrotizing retinitis with one year follow-up.

## Case report

**Fig. 1:**
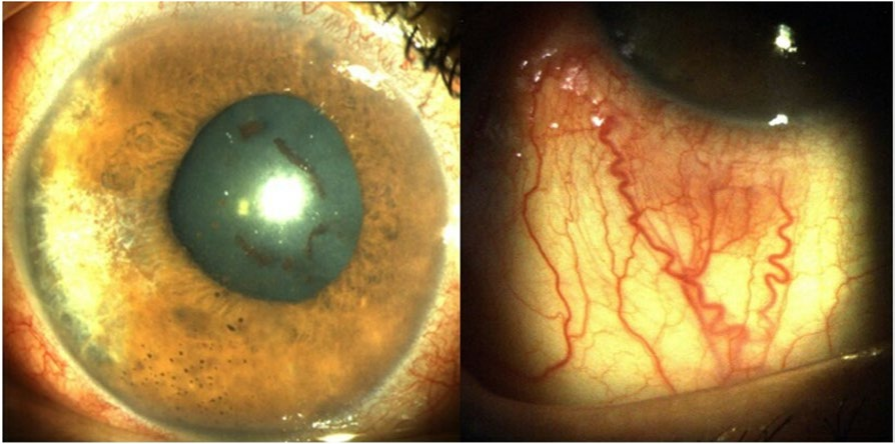
Right eye slit lamp anterior segment photographs showing, inferior and pigmented keratic precipitates with sectoral iris atrophy and broken posterior synechiae. The presence of a nodular perilimbal regular conjunctival lesion

**Fig. 2:**
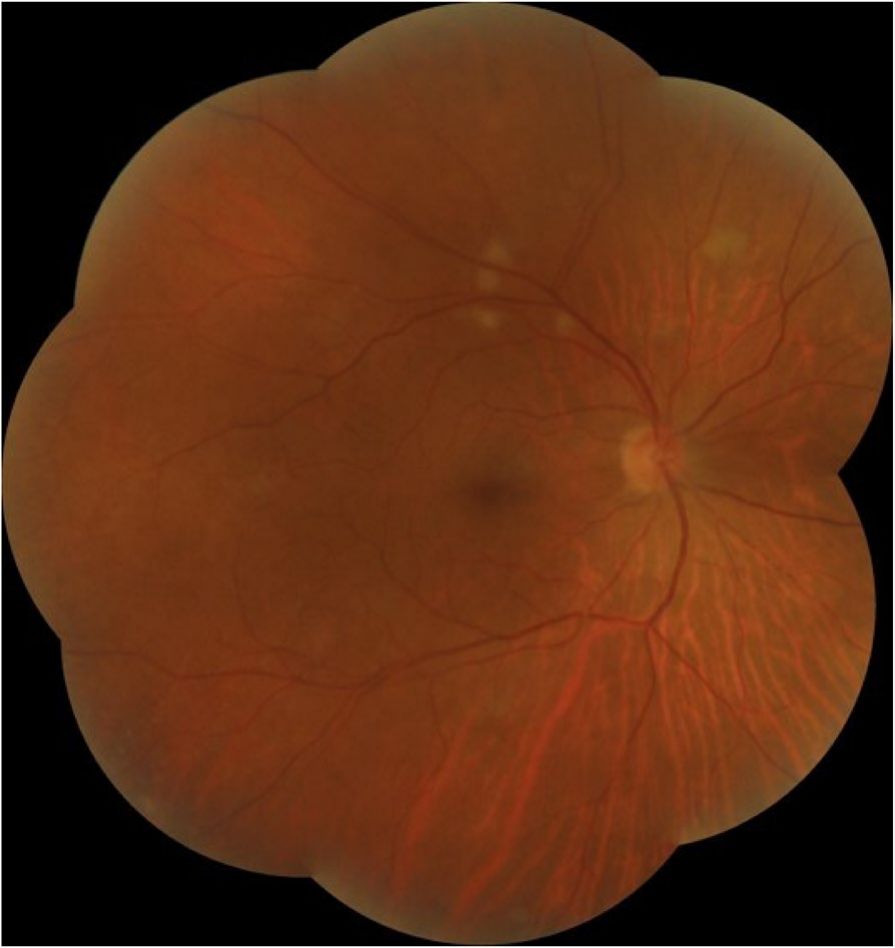
Color fundus montage photograph showing multiple focal retinitis with no retinal necrosis and normal retinal periphery

**Fig. 3:**
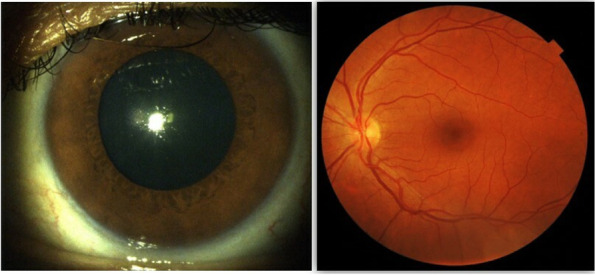
Left eye examination showing normal anterior segment and fundus photographs

**Fig. 4:**
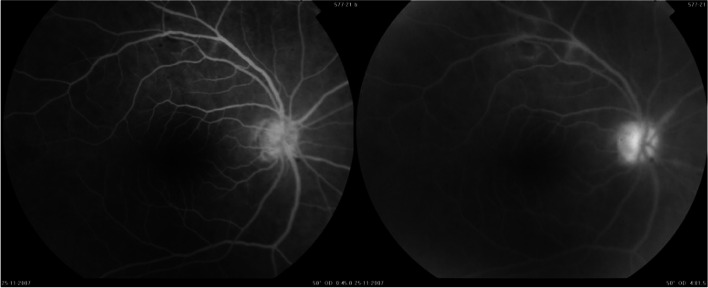
Fundus fluorescein angiogram of the right eye showing persistent hypo fluorescent lesions at early and late phase with hyperfluorescent borders and optic disc leakage

**Fig. 5:**
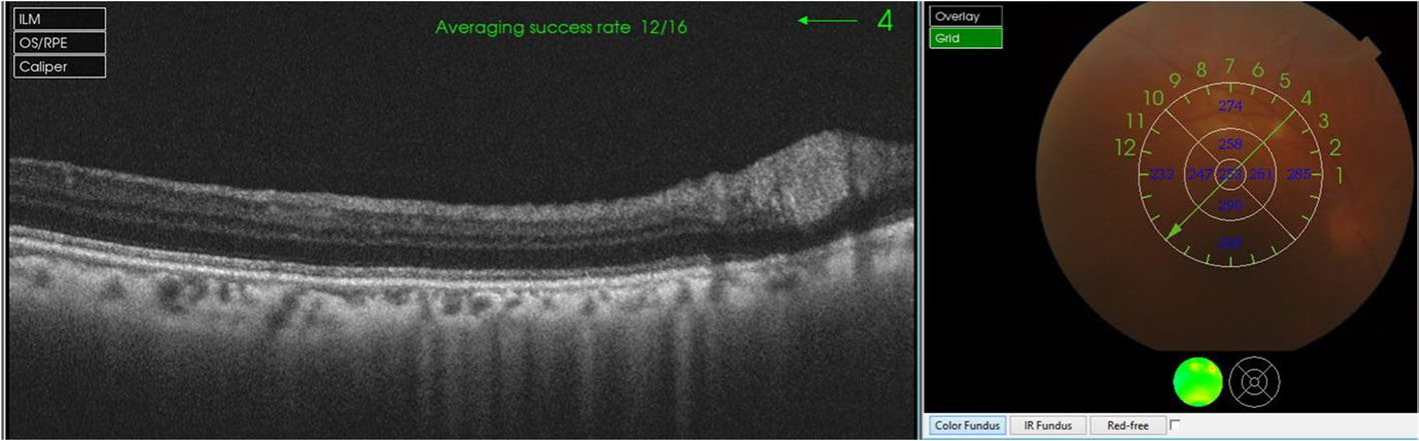
Swept source optical coherence tomography of the right eye showing hyper-reflectivity of the inner retinal layers corresponding to the retinal lesions

**Fig. 6:**
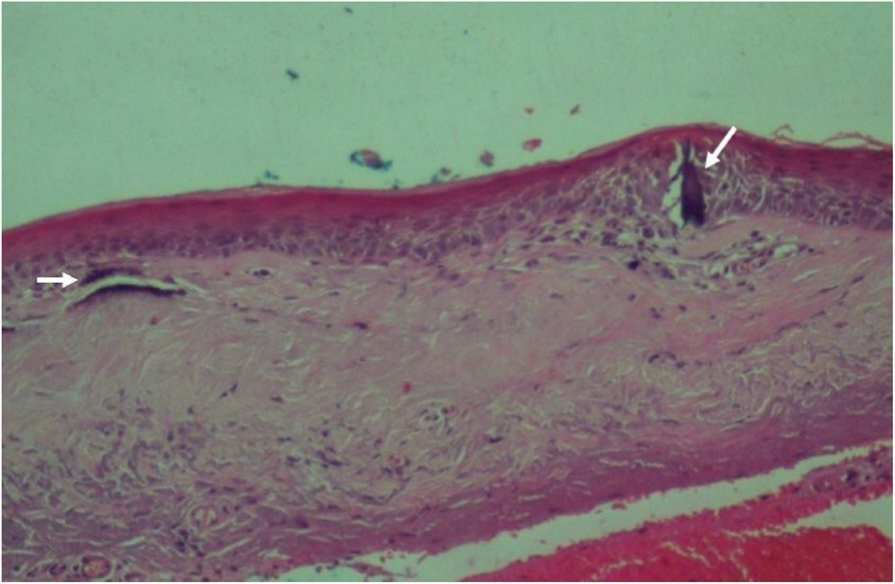
Histopathological examination of conjunctival nodule biopsy (× 200) revealing non-specific inflammatory remodeling with underlying calcifications (white arrows)

**Fig. 7:**
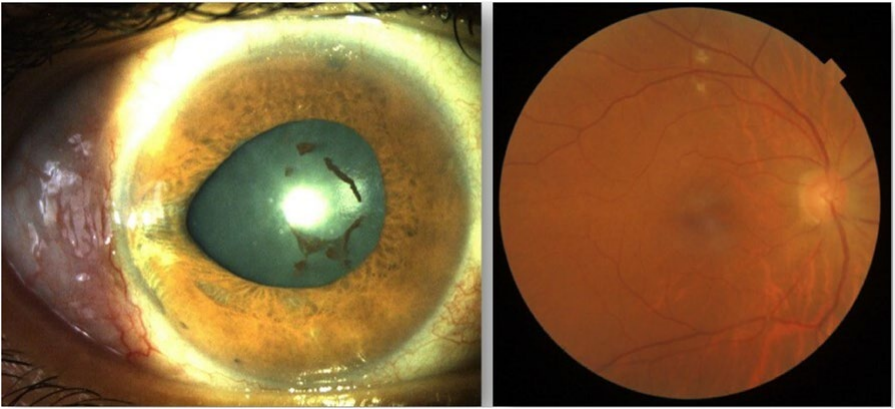
Right eye examination after 6 months of follow-up. Anterior segment photograph showing persisting sectoral iris distortion and atrophy and the disappearance of the keratic precipitates. Retinal fundus photograph showing the decreasing size of the retinal non necrotizing lesions

**Fig. 8:**
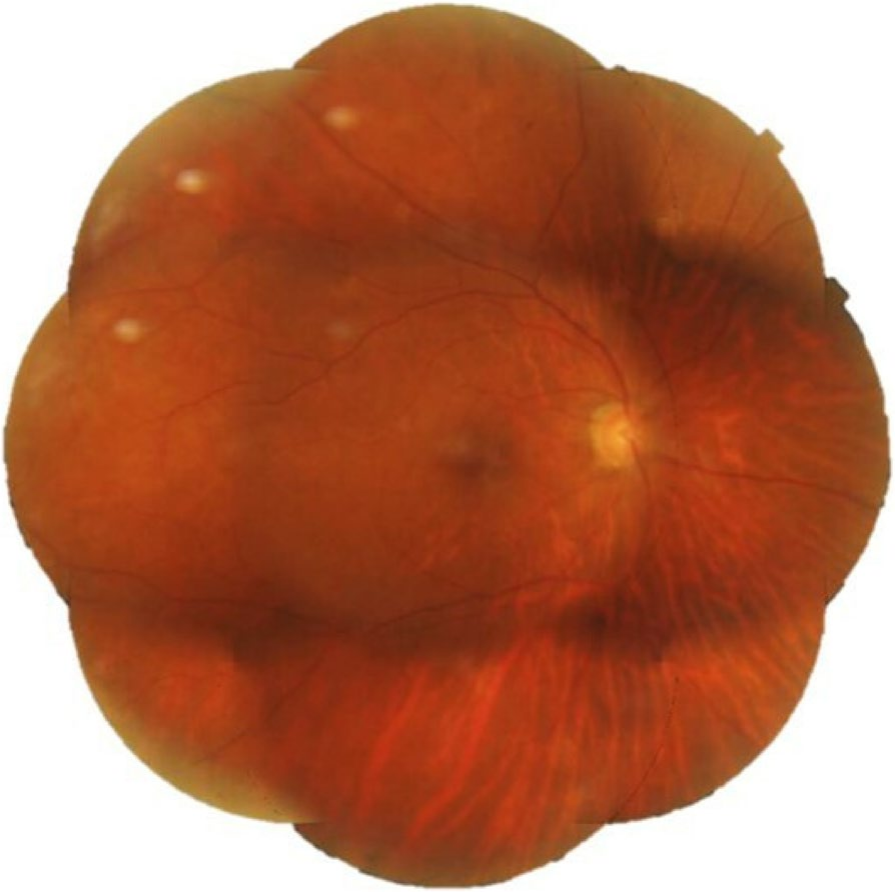
Color fundus montage photograph showing complete disappearance of the retinal non necrotizing lesions and some peripheral artifacts

**Fig. 9:**
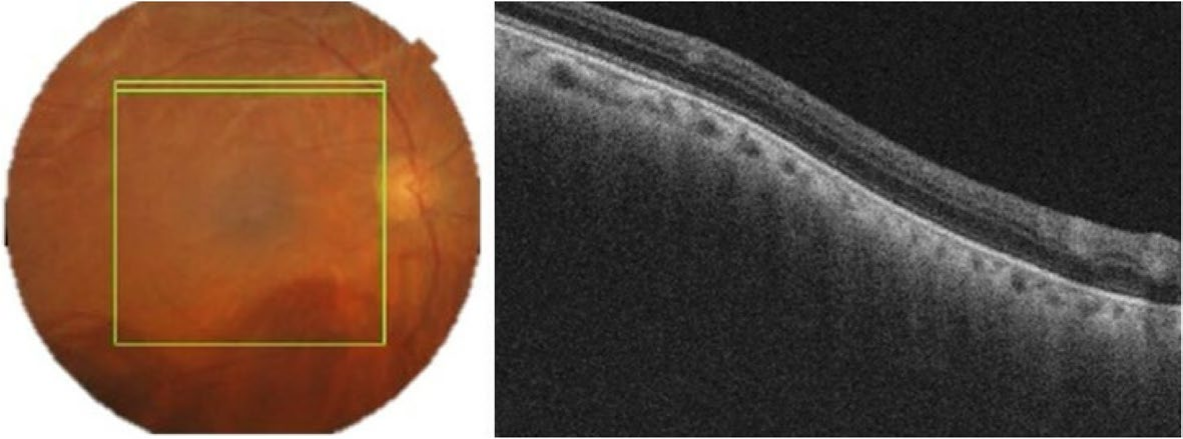
Swept source optical coherence tomography of the right eye revealing the absence of hyperreflectivity or inner retinal layer atrophy

A 52-year-old female with a past medical history of diabetes mellitus, presented with progressive pain, redness and vision loss of the right eye (OD). Her best corrected visual acuity (BCVA) in OD was 20/200 and 20/20 in the left eye (OS). Anterior segment examination of OD showed a perilimbal nodular conjunctival lesion, granulomatous anterior uveitis with sectoral iris atrophy (Fig. 1). Intraocular pressure was 28 mmHg in OD and 12 mmHg in OS. Fundus examination of OD disclosed multiple focal lesions of retinitis with no retinal necrosis (Fig. 2). OS examination was otherwise unremarkable (Fig. 3). In fluorescein angiography these focal retinal lesions appeared to be hypofluorescent in early and late phase with a peripheral hyperfluorescent border with significant optic nerve leakage (Fig. 4). Swept-source optical coherence tomography (SS-OCT) passing through these lesions showed hyper-reflectivity of the inner retinal layers (Fig. 5). Otherwise, macular SS-OCT was unremarkable. Histopathological examination of conjunctival nodular biopsy specimens showed a non-specific inflammatory remodeling with underlying calcifications (Fig. 6). Anterior chamber paracentesis (ACP) with an amount of 200 μl of aqueous humor (AH) was performed after an informed consent of the patient. Polymerase chain reaction (PCR) analysis came back negative for herpes simplex virus (HSV) types 1,2, CMV and positive for VZV. The patient was admitted and treated with intravenous acyclovir switched to oral valacyclovir (3 g/day) during 6 weeks with corticosteroid eye drops and control of ocular hypertension with association of beta blockers and carbonic anhydrase inhibitors eye drops. At six months of follow-up, she was maintained on antiviral therapy (valacyclovir 500 mg/ day). Her intraocular pressure was stabilized at 14 mmHg with absence of intraocular inflammation and an improvement of final BCVA to 20/40 in OD except the persistence of the retinal focal lesions (Fig. 7). They disappeared on the fundus examination and SS-OCT after one year of regular follow-up and at that moment we decided to stop the antiviral therapy with regular control (Figs. 8 and 9).

## Discussion

Case reports of NNHR have been described in five patients with positive test results for HSV or VZV in AH who had non-necrotizing retinitis consisting mainly of vasculitis, papillitis, or vitritis [2]. Other cases of non-necrotizing forms presenting as vasculitis and/or papillitis or panuveitis without retinal lesions were reported [3]. Albert et al. reported two cases of NNHR characterized by occlusive vasculitis with initial neovascularization. A herpetic etiology was confirmed by PCR analysis of ocular fluids: case 1 had a positive PCR for HSV1 on the AH while case 2 had a positive PCR for VZV on the vitreous sample [4]. Hazirolan et al. reported eight immunocompetent patients with NNHR. PCR analysis of vitreous samples confirmed the diagnosis: 63% of patients had HSV-1 DNA and 37% had VZV DNA [5]. Apart from NNHR, four cases of non-necrotizing herpetic vasculitis were also reported, and VZV was identified in the vitreous samples of two patients with PCR [6].

PCR was shown to be a powerful technique that allows detection of small quantities of DNA and RNA in ocular fluids. Various infectious agents can be detected with high specificity and sensitivity, including viruses. Results of PCR analysis of ocular fluids, being available within 24 to 48 h, have greatly improved the diagnosis of ocular viral infections, particularly those caused by human herpesviruses. AH analysis was contributory in 86.4% of patients with necrotizing viral retinopathies [3]. Bodaghi et al. found that the detection of HSV and/or VZV DNA by means of PCR in the AH was highly significant of recent productive infection in NNHR [2]. Our patient didn’t receive antiviral therapy before ACP. Indeed, antiviral drugs might decrease viral replication below the threshold of PCR sensitivity for viral detection [7]. A negative paracentesis could be repeated, especially in patients previously treated with antiviral therapy.

The viral retinitis described in our patient is different from most cases of atypical viral retinitis reported in the literature, but resembles the cases reported by Hazirolan et al., with retinal lesions that are smaller and focally situated at the posterior pole without progression with antiviral treatment [5].

The underlying reason for the variable clinical manifestations and severity of herpetic posterior uveitis is unknown but could be, in addition to pathogen-related factors, influenced by the variation in patients’ immune capacity. Bodaghi et al. hypothesized that necrotizing herpetic retinitis were due to an intracellular presence of the virus with subsequent cytopathic damage, whereas the NNHR were assumed to be associated with tissue damage based on immunological processes [2]. Wensing et al. suggested that NNHR occur in patients who have better functioning cellular immunity than patients with full-blown ARN [3].

Hazirolan et al. suggested that viral retinopathies might constitute a continuous spectrum of diseases, which clinical presentations depend on the patient’s immune status. Our patient, as well as theirs, with focal posterior non-necrotizing viral retinitis may be located at the starting point of the spectrum of herpetic retinopathies and constitute the mildest form of the disease [5]. However, more studies are required to test these hypotheses.

A remission was obtained in all cases after reaching the proper etiological diagnosis and initiating antiviral treatment. Oral acyclovir is mostly used for 6 weeks [8], but this period can be longer according to the disease response and the clinician practice [9]. Valacyclovir can also be used in the treatment of viral retinitis [10].

Albert et al. reported several recurrences after arrest of antiviral treatment, it was the first case of anterior uveitis recurrence after NNHR described in the literature [4]. Long-standing antiviral prophylaxis could be considered for such patients who present with recurrences of intraocular inflammation.

Future studies including a larger number of NNHR patients could reveal characteristics we were unable to identify here. The differential diagnosis of atypical viral retinitis is difficult clinically as it can mimic various other retinal conditions. In agreement with the previous studies, we propose viral analysis of ocular fluids in patients with retinitis, even in the absence of retinal necrosis, before initiation of immunomodulatory therapy and/or in those patients whose symptoms worsen while undergoing immunomodulatory treatments.

## Conclusion

Varicella zoster virus can cause a wide spectrum of clinical manifestations ranging from severe ARN to slow-progressing necrotizing and non-necrotizing types of inflammation. Non-necrotizing herpetic retinitis are currently underdiagnosed. PCR analysis of ocular fluids along with multimodal imaging could contribute to the confirmation and thus an earlier recognition of the diagnosis and the initiation of appropriate therapy.

## Data Availability

The data used in that case report is available from the corresponding author on reasonable request.
